# Evaluation and Management of Symptomatic Vasospasm following Endoscopic Endonasal Resection of Pediatric Adamantinomatous Craniopharyngioma

**DOI:** 10.1155/2020/8822874

**Published:** 2020-11-20

**Authors:** Dustin Hansen, Joaquin Hidalgo, Alan Cohen, Debraj Mukherjee, Susanna Scafidi

**Affiliations:** ^1^Department of Anesthesiology & Critical Care Medicine, Division of Pediatric Critical Care, Johns Hopkins University School of Medicine, Baltimore, MD, USA; ^2^Department of Neurosurgery, Division of Pediatric Neurosurgery, Johns Hopkins University School of Medicine, Baltimore, MD, USA

## Abstract

**Background:**

Cerebral vasospasm is a well-described pathology following subarachnoid hemorrhage and trauma in children; however, very few cases have been published following craniopharyngioma resection in children. Those that were published were associated with significant morbidity or mortality at hospital discharge. *Case Summary*. Here, we report the challenging clinical course of a pediatric patient who developed delayed cerebral vasospasm following craniopharyngioma resection. It was first noted on postoperative day 13. The patient was managed with induced hypertension, hypervolemia, and intra-arterial vasodilator therapy (nicardipine). This patient made a full recovery without new focal deficits at hospital discharge.

**Conclusion:**

In contrast to previously reported similar pediatric cases, this patient with cerebral vasospasm after craniopharyngioma resection made a full recovery without new focal neurologic deficits. To our knowledge, this is the first occurrence of a patient with this clinical course.

## 1. Introduction

Cerebral vasospasm is a well-known phenomenon following aneurysmal subarachnoid hemorrhage and trauma. While cerebral vasospasm is observed following pituitary tumor resections in adults, it remains a rare phenomenon [1, 2]. It has been described as a “reversible angiopathy” which presents with clinical signs of stroke or “radiographic features of cerebrovascular stenosis” [3]. The pathophysiology of this reaction is not well understood although several mechanisms have been proposed. Specifically, it is thought to be due to direct handling of the vessels (with spasm clinically manifesting in the first 3 days postsurgery), chemical effects of local craniopharyngioma fluid (manifesting between days 4–7 postsurgery), subarachnoid blood spillage, fluid and electrolyte shifts secondary to hypothalamic-pituitary axis (HPA) instability, or a combination thereof (clinically evident between days 4 and 14 postsurgery) [4, 5].

To date, there are only two previously reported cases of pediatric patients who developed cerebral vasospasm following craniopharyngioma resection, noted on days 3 and 5 [2, 6]. Both of these cases resulted in severe adverse outcomes (one patient died, while the other survived with hemiplegia). Here, we report a case of a delayed cerebral vasospasm following craniopharyngioma resection in a pediatric patient with subsequent good neurologic outcome.

## 2. Case Report

A 14-year-old female patient presented with new onset headache and left monocular hemianopia. MRI of the brain revealed a suprasellar calcified and cystic mass which was concerning for craniopharyngioma. The patient was evaluated by endocrinology and subsequently diagnosed with central hypothyroidism, diabetes insipidus (DI), presumed central hypogonadism (Tanner stage 1), and growth hormone deficiency (3rd percentile of height for age). The patient subsequently underwent an endoscopic endonasal trans-sphenoidal approach for resection of the craniopharyngioma. A lumbar drain was placed to decrease risk of cerebrospinal fluid (CSF) leak. Her immediate postoperative course was uncomplicated, and she was started on 50 mg/m^2^/day of hydrocortisone, a vasopressin infusion of 4 milliunits/kg/hour and continued on her previous levothyroxine of 50 mcg/day. Mean arterial blood pressure (MAP) was maintained greater than 80mmHg for optic apparatus perfusion. She was discharged from the Pediatric Intensive Care Unit (PICU) on postoperative day 3 to the general pediatric ward.

**Figure 1:**
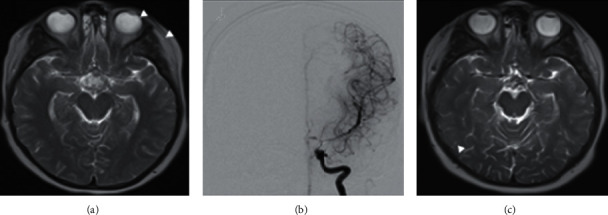
(a) Postoperative axial T2 sequence magnetic resonance of the brain evidencing reduced flow void diameters of the middle and anterior cerebral arteries surrounding the surgical resection cavity (postoperative day 13); (b) anteroposterior view digital subtraction angiography of a left ICA contrast injection evidencing severe vasospasm of the terminal portion of the ICA (arrow), proximal anterior, and middle cerebral artery (postoperative day 17); (c) postoperative axial T2 sequence magnetic resonance of the brain demonstrating improved flow void caliber of the terminal: internal carotid, middle, and anterior cerebral arteries (3 months postoperatively).

On postoperative day (POD) 13, the patient acutely developed confusion and was unable to follow commands, which prompted readmission to the PICU. The patient also had left eye and head deviation as well as diffuse weakness. Serum sodium at that time was 136 mEq/L, while other labs including metabolic panel and complete blood count were unremarkable. Ultrafast MRI (T2 sequence and DWI) of the brain showed new acute infarction within the water-shed distribution at the left anterior cerebral artery (ACA)/middle cerebral artery (MCA) border zone as well as small foci of acute infarction in the right paramedian frontal lobe and the right ACA/MCA border zone. In addition, imaging demonstrated diffuse narrowing of the terminal internal carotid arteries (ICAs) and of the proximal MCAs and ACAs, which was suggestive of vasospasm (Figure 1(a)). Continuous EEG was negative for seizure activity. Triple H therapy (hypertension, hypervolemia, and hemodilution) and nimodipine were initiated. Within approximately 12 hours, the patient began to recover speech and strength throughout. 24 hours following initiation of induced hypertension and hypervolemia (POD14), the patient had nearly returned to neurologic baseline, except for an intermittent left monocular visual field deficit on bedside examination, consistent with vasospasm noted on transcranial Doppler (TCD) studies.

Despite continuous hydration, normonatremia (serum Na = 135–145 mEq/L), and vasopressin drip, TCD on POD 14–16 showed velocities >100 cm/sec consistent with vasospasm. Clinically, the patient was not symptomatic when hydration status was maintained (input > output) and mean arterial blood pressure (MAP) remained >80 mm Hg (which is above the age-specific norm by 20 mmHg). On POD day 17, the patient had an acute, complete loss of vision in the left eye, which prompted surgical exploration and local papaverine application in the suprasellar space. Just prior to and after surgery, she had cerebral angiograms which demonstrated severe, diffuse vasospasm involving the anterior and posterior circulations bilaterally, moderately responsive to intra-arterial injections of nicardipine postoperatively. The left carotid circulation was the most severely affected (Figure 1(b)).

**Figure 2:**
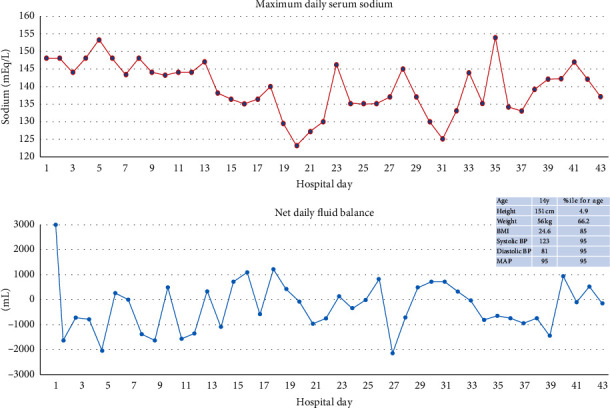
Daily net fluid balance and serum sodium values during the acute period of hospitalization.

Following the cerebral angiogram, the patient continued to have a brisk diuresis despite continuous infusion of vasopressin (peaked at 37 mU/kg/hr) and aggressive hydration (Figure 2) with fluctuating neurological examinations. A decision was made to maintain a MAP goal >90 mm Hg in the first 24 hours postsurgical exploration, and this required continuous infusions of vasoactive agents (phenylephrine and norepinephrine). The nimodipine had to be discontinued due to hypotension following administration.

The patient continued to have evidence of vasospasm on TCDs until POD 34 when TCD normalized. By this time, the patient had been weaned off all vasoactive medications and was transitioned to intranasal DDAVP with good response.

At discharge from the hospital, she did not have clinical deficits from her right frontal corona radiata infarct. Repeat MRI at approximately three months after vasospasm showed postsurgical changes with normalized diameter of the ACA/MCA and without evidence of new restricted diffusion (Figure 1(c)). On follow-up evaluation at six months after operation, the patient had returned to school and continued to do well with stable neurologic exam.

## 3. Discussion

To our knowledge, this is the third reported case of cerebral vasospasm in a pediatric patient following craniopharyngioma resection [2, 6]. However, this is the first case where the vasospasm occurred nearly 2 weeks after initial resection and the first reported case where despite severe vasospasm, no new long-term neurological deficits occurred. As in previous cases in adults, the etiology of vasospasm in this patient is unclear. The development of vasospasm on POD 13 was within the window of 3–19 days, as was noted in a case series of 6 adult patients who developed vasospasm after craniopharyngioma resection [7]. The patient's course was complicated by severe DI with rapid fluid and electrolyte shifts, both of which could have contributed to the etiology, timing, and severity of symptoms. In previous adult cases, it appeared that patients uniformly responded well to induced hypertension and hypervolemia [8]. Nimodipine has been shown to be beneficial for patients with aneurysmal subarachnoid hemorrhage and subsequent cerebral vasospasm. In our patient, the administration of nimodipine resulted in profound blood pressure fluctuations and recurrence of neurological symptoms.

The diagnosis of vasospasm may be easily overlooked and may result in delay of therapy and increased morbidity and mortality. In this patient, diagnosis was hinted at by the clinical presentation and imaging using ultrafast MRI, which included T2 sequence of the brain. MRI showed narrowing of the circle of Willis flow voids, prompting further vascular imaging and confirmation of the diagnosis. In the setting of acute neurological deterioration after craniopharyngioma resection, the diagnosis of cerebral vasospasm should be in the differential and not overlooked in order to establish prompt diagnosis and appropriate therapy.

## 4. Conclusion

Cerebral vasospasm should be considered as a potential complication following craniopharyngioma resection in pediatric patients. A high index of suspicion, prompt evaluation, and intervention are essential for improved outcome, especially considering the fact that development of cerebral vasospasm following tumor resection is associated with high morbidity and mortality.

## Data Availability

The data used to support the findings of this study are available from the corresponding author upon request.
